# Update on therapeutic approaches in ANCA-associated vasculitis

**DOI:** 10.1016/j.ero.2025.11.005

**Published:** 2025-11-28

**Authors:** David Jayne

**Affiliations:** Department of Medicine, University of Cambridge, Cambridge, UK

## Abstract

Standard-of-care therapy for antineutrophil cytoplasmic antibody-associated vasculitis (AAV) remains dependent on glucocorticoids (GCs) and immune suppression, especially rituximab, but patient outcomes are directly affected by only partial efficacy in controlling active disease, high levels of toxicity, and risks for subsequent relapse. GC dosing has been optimised in randomised controlled trials and the complement inhibitor, avacopan, introduced as an alternative to GCs. Improved understanding of the inflammatory and immune dysregulation in AAV has provided many cytokine, complement, and B cell targets, which are driving new drug development.

## INTRODUCTION

Long-term outcome studies from the European Vasculitis Trials have highlighted increased mortality, at all ages, and occurrence of end-stage kidney disease in 20% of patients with antineutrophil cytoplasmic antibody (ANCA)-associated vasculitis (AAV) [1,2]. Furthermore, almost all patients develop chronic morbidity due to organ dysfunction, treatment toxicity, and impaired quality of life. There is a need to increase the speed, quality, and proportion of patients achieving remission; to reduce the toxicity of therapy; and to reduce longer-term relapse rates, which exceed 50% at 5 years [3]. The current standard-of-care treatment is the combination of glucocorticoids (GCs) with either rituximab or cyclophosphamide. Although GC dosing has been standardised by the PEXIVAS and LOVAS trials, GC toxicity affects almost all patients and contributes to adverse outcomes [4,5]. Rituximab was not shown to be safer to cyclophosphamide in the RAVE trial for remission induction, and its use is associated with a high relapse risk unless it is continued long term [6,7]. But, larger observational studies have shown higher rates of recurrent infection with rituximab than other therapies and the development of secondary immunodeficiency in over 40% of patients [8,9].

## COMPLEMENT INHIBITION

The ability of the complement fragment C5a to attract and prime neutrophils for activation made it an obvious target for newer therapies given the neutrophil predominance and role of ANCA in pathogenesis [10,11]. Avacopan is an oral, C5a receptor 1 inhibitor that was shown in the ADVOCATE trial to increase rates of sustained remission at 12 months when compared with a GC-tapering regimen, to reduce relapse risk, reduce GC toxicity, and improve recovery of quality of life and kidney function [12]. EULAR recommendations favour avacopan as an alternative to GCs for patients in whom GC toxicity is a particular risk, but in real-world use, it has also been used for patients with acute kidney injury and refractory disease [13,14]. However, avacopan did not increase remission rates, and only 70% of patients showed remission by 6 months, with fewer by 3 months [12]. No pharmacodynamic studies have been performed in patients to validate current avacopan-dosing strategies. Development of vilobelimab, a monoclonal anti-C5a antibody, has been halted despite successful phase II studies in AAV (NCT03712345). The search for other complement inhibitors has been supported by pathologic evidence of C3 convertase activity, C5b pathway activation, along with classical complement pathway activation in some patients, suggesting a broader approach to complement inhibition may be more effective [15]. Recent evidence has shown associations of plasma and urine-activated factor B levels with disease activity, supporting the validity of this target [16]. Moreover, iptacopan, an oral factor B inhibitor already approved for paroxysmal nocturnal hemoglobinuria and IgA nephropathy, is in a phase II trial for AAV (NCT06388941) [17]. It has the potential risk, along with anti-C5 therapies, such as eculizumab, of infection with encapsulated bacteria, thus requiring immunisation or antibiotic prophylaxis.

## CYTOKINE INHIBITION

Interleukin (IL)-5 is a driver of eosinophil infiltration and tissue injury in eosinophilic granulomatosis with polyangiitis (EGPA), and its inhibition with mepolizumab led to approval for this indication [18]. A recent head-to-head trial, MANDARA, between mepolizumab and an anti-IL5 receptor monoclonal antibody, benralizumab, found no difference in the proportion achieving the primary end point of disease control with a prednisolone dose of <5 mg/d [19]. In a secondary end point more patients on benralizumab were able to fully withdraw GC and circulating eosinophil counts were lower. Both drugs achieved at least a 50% reduction in GC requirement and are supported by guideline statements for patients with EGPA with relapsing disease unable to reduce prednisolone to 7.5 mg/d [13]. A long acting form of mepolizumab, depemokimab, is being evaluated in the OCEAN trial (NCT05263934). These studies have excluded patients with active vital organ–threatening disease, but this subgroup is being recruited to the EMERGE trial conducted by the French Vasculitis Study Group. Genetic variants associated with IL-5 were discovered in a genome-wide association survey of patients with EGPA, along with thymic stromal lymphopoietin (TSLP), and tezepelumab, an anti-TSLP monoclonal antibody approved for eosinophilic asthma, is being tested for EGPA in the RACEMATE trial [20]. NS Pharma are conducting a phase II trial of a JAK1 specific antagonist NS-229 in EGPA (NCT06046222), JAK1 signalling influences several cytokine pathways relevant to EGPA including IL-6 and IL-7.

## B CELL TARGETING

Although rituximab is now established as the default therapy for induction and remission phases of AAV, it is only partially effective and has adverse effects outlined above. The cytokines required for B cell development and activation, B cell Activdating Factor (BAFF) and A Proliferation Inducing Ligand (APRIL), are increased in patients with AAV, and BAFF signalling in tissue between switched memory B cells and T follicular helper cells plays an important role in driving relapsing disease [21]. The BAFF inhibitor belimumab failed in the BREVAS trial, but this has been partly explained by concerns over trial design [22]. A synergy between rituximab and belimumab has been demonstrated in the BEAT-LUPUS trial in SLE and is being explored in AAV in the COMBIVAS trial [23]. Combined inhibition of both BAFF and APRIL with TACI receptor constructs, povitacicept and telitacicept, are in phase II trials in AAV, with some anecdotal data in refractory disease already published [24]. B cells in tissue are not fully depleted by rituximab, and this has encouraged investigation of more potent anti-CD20 antibodies such as obinutuzumab, recently successful for lupus nephritis in the REGENCY trial [25]. Obinutuzumab is already available for refractory AAV, and the OBIVAS trial is directly comparing the ability of obinutuzumab to deplete tissue B cells, with that of rituximab [26]. As a relapsing B cell–mediated autoimmune disease, anti-CD19 CAR-T holds the potential for long-term treatment-free remission. Following a proof of principle of this approach in an animal model, the first reports of success in patients are appearing [27,28]. The complexity of delivering autologous CAR-T therapy for a disease with severe inflammation presents challenges in trial design, and simpler approaches including allogeneic anti-CD19 CAR-T or bispecific anti-CD19:anti-CD3 T cell engagers, which can be administered without delay, may provide more practical alternatives. Following observational experience of rituximab in EGPA, a randomised trial comparing rituximab with conventional glucocorticoid dosing with or without cyclophosphamide, found no improved response with rituximab [29]. The potential benefit of sequential rituximab and anti-IL5 therapy has been reported [30].

## CONCLUSIONS AND FUTURE DIRECTIONS

While clinical epidemiology has better defined the consequences of AAV for patients and the need for better therapies, there has been rapid acceleration of dug development in this area. We can look forward to wider use of complement inhibition with direct benefits of less GC use and more potent anti-B cell strategies that may simplify longer-term remission strategies. T cell activation is a consistent feature of AAV and has not been specifically targeted although there is some preliminary data with the IL-12/23 inhibitor ustekinumab, while abatacept—CTLA4Ig—failed to show benefit in the ABROGATE trial of nonsevere granulomatosis with polyangiitis [31,32]. There is experimental evidence for control of vasculitis by cathepsin C inhibition suppressing neutrophil activation and an ongoing phase II trial of a claudin 1 monoclonal antibody, lixudebart, as an antifibrotic agent in AAV with acute kidney injury [33,34].

## Funding

This research was supported by the NIHR Cambridge Biomedical Research Centre.

## Competing interests

DJ reports consulting or advisory for AstraZeneca Pharmaceuticals LP, GSK, Alentis Therapeutics AG, Novartis, Vifor Pharma Switzerland SA, and Otsuka Pharmaceutical Co Ltd; board membership of and equity or stocks in Aurinia Pharmaceuticals Inc; equity or stocks in Alentis Therapeutics AG; funding grants and speaking and lecture fees from Vifor Pharma Switzerland SA; and speaking and lecture fees from Otsuka Pharmaceutical.

## Patient consent for publication

Not applicable.

## Ethics approval

Not applicable.

## Provenance and peer review

Not commissioned, externally peer reviewed.

## CRediT authorship contribution statement

**David Jayne:** Writing – review & editing, Writing – original draft, Conceptualization.
